# Advancements in genetic research and RNA therapy strategies for amyotrophic lateral sclerosis (ALS): current progress and future prospects

**DOI:** 10.1007/s00415-025-12975-8

**Published:** 2025-02-26

**Authors:** Paola Ruffo, Bryan J. Traynor, Francesca Luisa Conforti

**Affiliations:** 1 Neuromuscular Diseases Research Section, National Institute on Aging, National Institutes of Health, Bethesda, MD USA; 2 Medical Genetics Laboratory, Department of Pharmacy, Health and Nutritional Sciences, University of Calabria, Rende, Italy; 3 Department of Neurology, Johns Hopkins University Medical Center, Baltimore, MD USA

**Keywords:** Amyotrophic lateral sclerosis, Non-coding RNA, Next-generation sequencing, Gene-targeted therapies

## Abstract

This review explores the intricate landscape of neurodegenerative disease research, focusing on Amyotrophic Lateral Sclerosis (ALS) and the intersection of genetics and RNA biology to investigate the causative pathogenetic basis of this fatal disease. ALS is a severe neurodegenerative disease characterized by the progressive loss of motor neurons, leading to muscle weakness and paralysis. Despite significant research advances, the exact cause of ALS remains largely unknown. Thanks to the application of next-generation sequencing (NGS) approaches, it was possible to highlight the fundamental role of rare variants with large effect sizes and involvement of portions of non-coding RNA, providing valuable information on risk prediction, diagnosis, and treatment of age-related diseases, such as ALS. Genetic research has provided valuable insights into the pathophysiology of ALS, leading to the development of targeted therapies such as antisense oligonucleotides (ASOs). Regulatory agencies in several countries are evaluating the commercialization of Qalsody (Tofersen) for *SOD1*-associated ALS, highlighting the potential of gene-targeted therapies. Furthermore, the emerging significance of microRNAs (miRNAs) and long RNAs are of great interest. MiRNAs have emerged as promising biomarkers for diagnosing ALS and monitoring disease progression. Understanding the role of lncRNAs in the pathogenesis of ALS opens new avenues for therapeutic intervention. However, challenges remain in delivering RNA-based therapeutics to the central nervous system. Advances in genetic screening and personalized medicine hold promise for improving the management of ALS. Ongoing clinical trials use genomic approaches for patient stratification and drug targeting. Further research into the role of non-coding RNAs in the pathogenesis of ALS and their potential as therapeutic targets is crucial to the development of effective treatments for this devastating disease.

## Introduction

“Are we ready for genome-wide association studies (GWAS)?” This question was raised in 2006 to introduce topics about GWAS [76]. Since then, more than 9000 studies have been published, which showed the discovery and replication of many novel loci for various phenotypes, highlighting the success of GWAS and lending aid to the common disease–common variant (CDCV) idea. The CDCV hypothesis proposes that a significant ratio of phenotypic variance in a population is due to common variants, suggesting that vulnerability for a given trait is primarily due to common variants. Typically, the variants retained in standard genotyping arrays for GWAS are single nucleotide polymorphisms (SNPs) with minor allele frequency (MAF) greater than 1%. They are thought to represent most of the genetic risk for a given disease attributed by common variants. However, despite the success of GWAS in defining robust risk factors for complex diseases, this approach only captures a fraction of the heritability of these disorders, even when substantial sample sizes were analyzed [50].

**Fig. 1:**
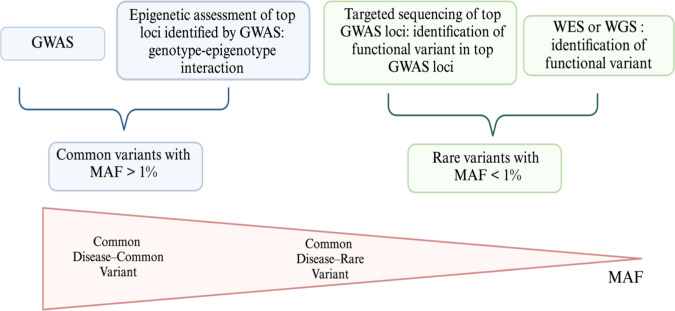
Decode neurodegenerative diseases by combining the CDCV and CDRV hypotheses. GWAS is used to identify common variants with MAF more significant than 5%. The common variants identified are likely not causative but in linkage disequilibrium with causal variants (common disease–common variant – CDCV- hypothesis). NGS approaches are now being applied by performing targeted resequencing of top loci identified by GWAS or whole-exome sequencing in multiple affected cases to identify potential causal variants (common disease–rare variant–CDRV–hypothesis). Triangle indicates gradient change in MAFs from greater than 5% to less than 1%

Parallel to the common disease–common variant hypothesis, the common disease–rare variants (CDRV) idea has been proposed to explain the missing heritability. The CDRV hypothesis states that considerable rare variants (with MAF < 1%) may underlie susceptibility to common diseases. SNPs alone (whether common or rare) will not account for all the genetic heritability, and there are other types of variants (structural variations, etc.) that are at play. Nevertheless, the accelerated expansion of next generation sequencing and the continued use of GWAS approaches in elucidating the genetic etiology of complex disorders suggests that the views are not mutually exclusive (Fig. 1).

Apart from decoding the genetic etiology of complex diseases, next-generation sequencing (NGS) approaches also increasingly play an essential role in disease diagnosis. NGS approaches are already showing promising results in diagnostic settings for conditions with a clear Mendelian inheritance, as shown for Charcot-Marie-Tooth disease. However, in diseases with considerable clinical and genetic heterogeneity (complex disorders), the identification of a putative mutation needs to be validated, either by conducting segregation analyses in independent cohorts or by association testing in large cohorts of various ethnic heritage, before clinical diagnosis or pathogenicity due to a precise mutation can be verified.

## NGS advancements in neurodegeneration and ageing research

The development of NGS has revolutionized how genetic research is conducted, allowing: the analysis of entire genomes (whole genome sequencing) [64]; specific loci or selected candidate genes Targeted Sequencing [84], or sequencing of exons of all coding genes (whole exome sequencing) [8]. Unlike first-generation sequencing, also named Sanger sequencing, which took many years and cost billions of dollars to sequence the first diploid human genome, the NGS platform can generate the same genomic sequence in just 1 week at a drastically lower cost [77]. More importantly, NGS technology has enabled the identification of rare variants with large effect sizes, including the unmasking of missense or nonsense single-base substitutions, as well as small insertions or deletions, which have crucial implications in risk prediction in the diagnosis and treatment of age-related diseases [28].

Ageing is the irreversibly progressive decline in physiological function, ultimately leading to age-related diseases. Among these various conditions encountered during aging, neurodegenerative disorders (NDs) and their associated cognitive deficits are prevalent among older populations, impacting their healthy lifespan and quality of life. Neurodegeneration is an intricate brain disorder that is not yet fully comprehended.

**Fig. 2:**
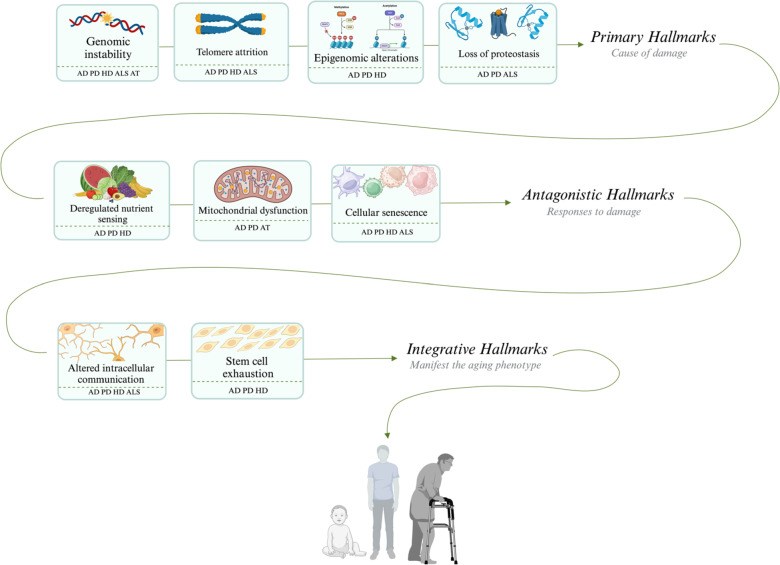
Nine hallmarks of aging seen in the main neurodegenerative diseases. *AD* Alzheimer disease, *ALS* amyotrophic lateral sclerosis, *AT* ataxia telangiectasia, *HD* Huntington disease, *PD* Parkinson disease. (Created with BioRender.com)

Ageing biomarkers are the most significant risk factors for neurodegeneration [88]. Nine crucial ageing hallmarks process have been identified in recent years [31, 43], each associated with the pathogenesis of at least one NDs. These nine-biological hallmarks have been broadly organized into primary, antagonistic, and integrative. Primary hallmarks of ageing enclose genomic instability, epigenetic alterations, telomeric attrition, and the loss of proteostasis. Antagonistic hallmarks refer to compensatory responses to primary damage associated with ageing, including mitochondrial dysfunction, cellular senescence, and the downregulation of nutrient sensing. Integrative hallmarks result from cumulative damage of primary and antagonistic hallmarks, including stem cell exhaustion and altered intercellular communications (Fig. 2) [31].

The prevalence of NDs among older populations is so common that disease-free brains are rare. Brain ageing might be a scale of neurodegeneration progression, and human genetic and environmental factors may act as determinants for the onset and progression of these disorders [88].

Due to neurodegeneration is among the most prevalent age-related diseases, which indicates the presence of a link between NDs and ageing-related modifications that occur in the brain microenvironment, such as genomic instability, epigenetic modifications, and the loss of proteostasis. Although ageing is known to be a significant risk factor for NDs, the precise mechanisms through which ageing is associated with neurodegeneration have not yet been identified. Molecular studies have identified that proteins like α-synuclein, phosphorylated tau, and Aβ aggregate abnormally with ageing; however, it is not confirmed whether they are associated with cognitive impairment [22]. Some other studies revealed that early-life developmental defects of the brain are associated with ND development risk; in that case, cognitive impairment might occur lately [15].

### Amyotrophic lateral sclerosis (ALS)

Among these, of particular interest for this review is Amyotrophic Lateral Sclerosis (ALS)—a neurodegenerative disease caused by the progressive loss of motor neurons resulting in weakness and paralysis of voluntary muscles. Although substantial research progress has been made, the etiopathology of ALS is mainly unknown [69]. The incidence of this disease has been reported to be approximately 1.5–2.5 per 100,000 people each year [74]. with an higher incidence of ALS in men than in women, with an approximate ratio of 1.5:1 [27, 30, 42], but this gender ratio decreases to 1:1 with age (more apparent after the age of 70 years) [30, 53, 42].

ALS is considered a complex genetic disorder with a Mendelian inheritance pattern in some instances but with no discernible family history in the rest. The disease occurs in sporadic (sALS, about 90%) and familial forms (fALS, about 10%). About 40–55% of cases with a familial background are due to pathogenic mutations in genes coding for *SOD1* (Superoxide Dismutase 1, OMIM *147,450) [67], *FUS* (Fused in Sarcoma, OMIM *13,707) [37], *TARDBP* (TAR DNA-binding protein 43, OMIM *605,078) [73] and a *C9Orf72* (hexanucleotide expansion on chromosome 9 in Open Reading Frame 72, OMIM *614,260) [9]. Most cases of sALS do not seem to have a clear genetic cause. However, de novo mutations—mutations that occur spontaneously rather than being inherited—have been found in genes such as *FUS*, *SOD1*, *SPTLC1* and several other genes related to ALS. The exact frequency of these de novo mutations in sALS is still unclear, but research suggests they may play a role in a subset of cases, particularly among patients with an earlier onset of the disease.

A study identified a de novo missense mutation in the *FUS* gene (c.1561C > T, p.R521C) in a patient with early-onset ALS, underscoring the significance of de novo mutations in sALS [11]. Another study revealed that *FUS* mutations are the most common genetic cause of early-onset ALS, with de novo mutations found in 43% of patients aged under 35 years [32]. Furthermore, de novo mutations in the *SOD1* gene have also been reported as a contributing factor to ALS, reinforcing the impact of spontaneous genetic changes on the disease [1, 57]. An autosomal dominant inheritance characterizes a familiar form of ALS. Rarely can it be transmitted as an X-linked or recessive trait [16]. The age-at-onset of fALS is approximately ten years earlier than sALS. However, otherwise, fALS and sALS are clinically indistinguishable, with both having similar disease progression and neuropathologically, having similar patterns of neuronal loss and inclusions. Due to the similarities in disease manifestation, many hypothesize that fALS and sALS may share a common pathogenic mechanism [3].

**Fig. 3:**
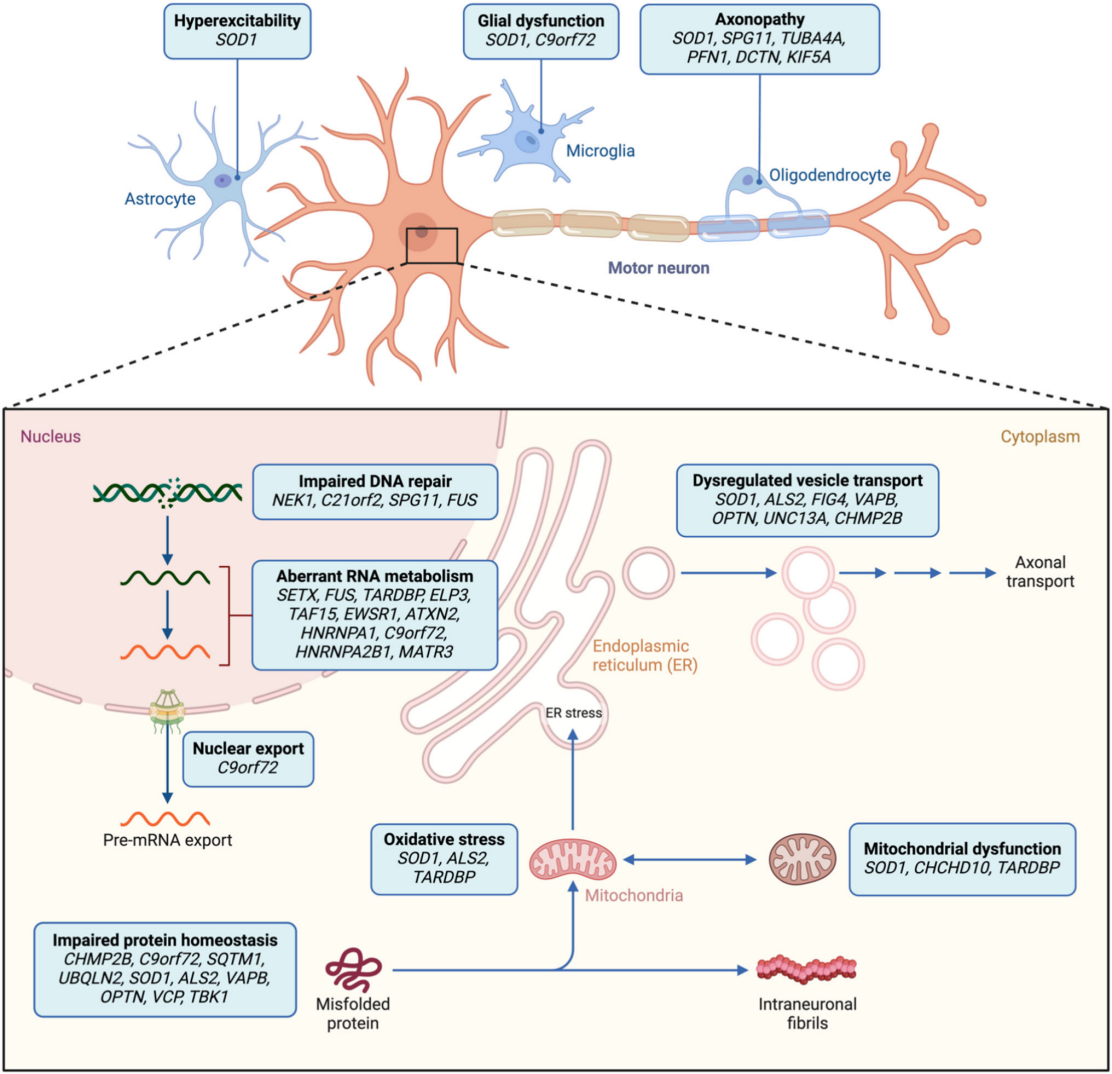
Pathophysiology of ALS. Mutations in various genes linked to ALS can harm motor neurons through multiple interconnected pathways. *SOD1* has been extensively studied and is associated with many mechanisms, while the effects of mutations in genes like *ALS3* and *ALS7* are unclear. RNA metabolism and protein homeostasis abnormalities connect several ALS-causing genes to neuronal damage. Mitochondrial dysfunction can arise from *CHCHD10* mutations or secondary respiratory chain deficiencies due to protein aggregates from other ALS mutations, increasing oxidative stress. ALS also directly affects neuronal function and glial cells, with neuronal hyperexcitability and axon dysfunction implicated. These mechanisms interact, complicating ALS pathophysiology

Studies of the pathophysiology of ALS conducted in cellular and animal models expressing mutant ALS-causing genes have enabled the discovery of key pathogenetic mechanisms relevant to the disease process. Moreover, with the application and use of new technologies and approaches, it has been possible to map genetic variants on the main pathogenetic mechanisms relevant to all cellular compartments of motor neurons (Fig. 3). These mechanisms include RNA metabolism and transport, protein aggregation, and the prion-like properties of the aggregating proteins, the role of stress granules formation, the perturbations of the mitochondrial function, inflammation, microglia activation, astroglia contribution to neurodegeneration, excitotoxicity, and oxidative stress as crucial factors in the pathogenesis of ALS. Because of their seemingly disparate function, studies on ALS-related genes other than the major causative genes have been scarce, and their conclusions are uncertain [25].

The only pathological feature that is observed to be present in both fALS and sALS, is the presence of mutant protein aggregates (*TDP-43, FUS*) in specific cytoplasmic inclusions in affected motor neurons [59, 79, 79]. It is, therefore, not clear whether the significant molecular mechanisms related to ALS pathogenesis identified in models expressing ALS-related gene mutations are also at work in sALS and the degree of shared pathogenesis is unclear. Other outstanding pathomechanisms uncertainties related to changes in RNA metabolism and transport, aggregation of proteins with prion-like properties, mitochondrial dysfunction due to the interaction of mutant proteins with specific mitochondrial proteins, e.g., *VDAC1* [47] or stress granule formation [21] remains open. Answering these questions will have a substantial impact on future research in ALS as it would help shape our understanding of which cellular pathways and/or processes can be targeted for therapeutic development.

### Role of genetics in ALS therapy development

Except for Riluzole (6-(trifluoromethoxy)−2-aminobenzothiazole), which was the first drug available for patients with ALS, and Edaravone (3-methyl-1-phenyl-2-pyrazoline-5-one), which decreases oxidative stress and the rate disease progression [86], no treatments are available for this neurodegenerative disease that can effectively stop or reverse the disease progression. Specifically, Riluzole is the only approved treatment worldwide and is recommended as the first line of disease-modifying therapy according to the most updated guidelines [81]. A population-based outcome study conducted in the Irish ALS population over five years reported that Riluzole therapy reduced mortality rates by 23% and 15% at 6 and 12 months, respectively, with an approximate survival benefit of four months, although this effect diminished in prolonged follow-up [78]. Similarly, an analysis of the Riluzole dose-ranging trial showed that the treatment prolonged time in stage 4 compared to placebo (hazard ratio 0.55, *p* = 0.037 for 100 mg/day), suggesting a survival benefit in advanced ALS stages. No significant differences in survival were observed for patients transitioning from stages 2 or 3, underscoring the benefits of Riluzole in later stages [24]. The positive effect of this drug is thought to result from different actions as a sodium channel blocker acting as a neuroprotective drug [58]. Riluzole also acts as an anti-glutamatergic agent via glutamate release reduction, the hypofunction prevention of glutamate receptors, and the increase in glutamate uptake by activating glutamate transporters. However, this mechanism of action alone cannot explain the neuroprotective effect, as other N-methyl-D-aspartic acid- and alpha-amino-3-hydroxy-5-methyl-4-isoxazole propionic acid-receptor-blocking drugs like gabapentin have not shown any effect on survival [19]. The discovery and sequencing of more than 40 genes linked to ALS has provided researchers with an initial list of possible targets for gene therapy. The latter involves the delivery of genetic material to cells in order to introduce functional copies of dysfunctional genes, introduce trophic factors and other disease-modifying genes, or silence harmful gene expression using antisense oligonucleotides (ASOs), RNA interference (RNAi), or gene-editing technology [2]. In April 2023, FDA Grants Accelerated Approval for Qalsody (Tofersen) to treat ALS patients associated with a superoxide dismutase 1 (*SOD1*) gene mutation. Furthermore, regulatory agencies in many countries are also in the process of assessing this drug for marketing and is already available in the United States of America and Germany. Qalsody is an antisense oligonucleotide that targets *SOD1* mRNA to reduce the synthesis of *SOD1* protein [7]. The key observations leading to the development of Tofersen and other emerging anti-*SOD1* therapies are based on different insights: the neurotoxicity associated with mutant *SOD1* results from a dose-dependent gain-of-function mechanism, and a specific segment of the mutant *SOD1* polypeptide is critical for this neurotoxic effect to take place. The final approval was based on reduced plasma and CSF neurofilament light (NfL), a blood-based biomarker of axonal (nerve) injury and neurodegeneration [7, 71]. As well as changes in delta ALSFRS-R and pulmonary functions [71], critical indicators of disease progression and respiratory decline in ALS.

**Fig. 4:**
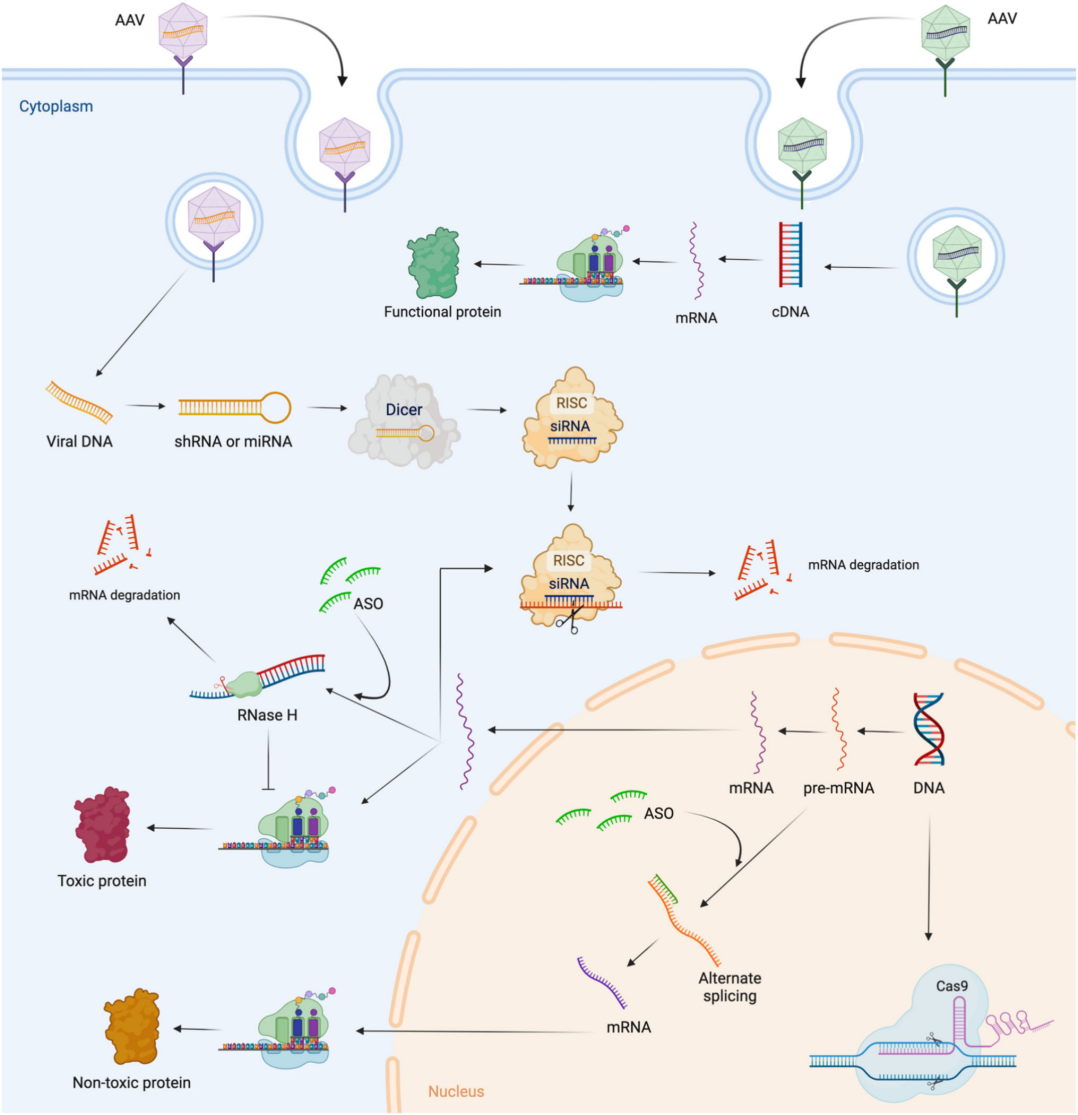
Possible approaches for gene therapy to treat amyotrophic lateral sclerosis. ASO are short synthetic oligonucleotides (~ 20 nucleotides). They bind to the targeted mRNA and either (i) cause the mRNA degradation by endogenous RNase H or (ii) block the mRNA translation. This eventually decreases the expression of specific proteins. In ALS, this strategy has been used to reduce the protein level of TDP-43, SOD1 of FUS protein level or to target C9orf72 RNA foci. SiRNAs are double-stranded RNAs that can bind argonaute proteins as part of the RISC complex, which finally leads to mRNA cleavage. Another option for functional replacement of a missing gene is gene (i.e., either mRNA or cDNA) delivery through viruses (e.g., adeno-associated viral vectors). This approach was utilized in SMA but needs more investigation in ALS. (Created with BioRender.com)

The complexity of ALS and the limited understanding of the pathogenetic mechanisms at play have hindered the progress of therapeutic development. In general, four approaches can be deployed to mitigate the toxic effects of etiological genes (Fig. 4): miRNAs or antisense oligonucleotide (ASO) for ablation of transcribed RNA from the gene. The administration of ASOs, which are synthetic nucleic acids that target/alter mRNAs, has shown promising results in treating pediatric neuromuscular disorders in children, such as spinal muscular atrophy (SMA) [68], Duchenne muscular dystrophy (DMD) [52] and ALS [54]. Another promising method is reducing excess mutant proteins by applying immune-mediated reduction. Interference with the transcriptional process using small molecules can also be a therapeutic tool. Last but not least, somatic cell mutagenesis to correct the gene mutation by returning it to the wild form. However, a key aspect of developing effective gene therapies is determining whether a mutation leads to a loss-of-function (LoF) or a gain-of-function (GoF) effect. LoF mutations result in a reduced or complete loss of the normal function of a gene, often due to deletions, truncations, or inactivating mutations [45]. In contrast, GoF mutations lead to an abnormal increase in gene activity, the production of toxic proteins, or novel functions that contribute to disease pathology [39]. This distinction is crucial because LoF mutations typically require strategies such as gene replacement or upregulation of compensatory pathways, whereas GoF mutations demand gene silencing approaches to prevent toxic effects [12].

Increased knowledge about the genetic profiles that protect or confer disease risk in patients with ALS will change how clinical trials are conducted and how therapy is prescribed. The most significant change will be the stratification of patient and control cohorts by genotype, increasing the success rate of clinical trials. Because ALS is a genetically heterogeneous and complex disease, a personalized medicine approach is emerging, whereby treatment is tailored to the specific mutation that causes disease in an individual patient. Thus, genetic screening for known variants or mutations will be integral to diagnosing, treating, and preventing ALS. Many advances have been achieved in the past 5 years, such as applying gene silencing for *SOD1* and *C9Orf72*, developing viable biomarkers for diagnosing patients with ALS with mutations in those genes, and evaluating the efficacy of potential treatments. More breakthroughs are expected when more mutations and genes are identified through these large-scale genetic studies. In addition to genetic screening, the Silence ALS platform represents a significant advancement in ALS treatment. It employs gene silencing technologies such as RNA interference (RNAi) and ASOs to target specific mutations associated with ALS. The platform aims to identify pre-symptomatic individuals who possess unique or rare ALS mutations and to develop personalized experimental ASO medications for their treatment. One pioneering project within the Silence ALS platform focuses on patients with rare pathogenic mutations in the *TARDBP* gene. The goal is to create individualized, allele-specific ASOs that target the mutated TDP-43 transcript. This effort builds upon the success of the initial Silence ALS program, which achieved the first-ever human dosing of an ALS patient with an allele-specific *TARDBP* ASO in October 2022. Another project under this initiative will develop a non-allele-specific ASO aimed at ultra-rare mutations in the *CHCHD10* gene, which are linked to ALS and FTD [44].

Different clinical trials have employed a genomic approach to select patients with specific drug-targetable gene mutations. Trial NCT01041222 was the first to use an intrathecally injected ASO to inhibit *SOD1* expression in *SOD1*-fALS mutation carriers. The results revealed a successful strategy, showing that the drug (ISIS 333611) was well tolerated [55]. A similar genetic stratification was applied in trial NCT00706147, where a genotype–phenotype homogeneous population of *SOD1*-fALS mutation carriers was used to test the safety, tolerability, and efficacy of Arimoclomol, a drug promoting the correct folding of proteins. The study demonstrated drug tolerability and safety but unfortunately, did not show therapeutic efficacy [4]. In trial NCT04494256, subjects with *ATXN2* expansion were enrolled to assess the safety, tolerability, and pharmacokinetic profile of BIIB105, an ASO designed to bind and degrade the *ATXN2* mRNA [68]. Part of this trial targets individuals with medium-length CAG expansions in *ATXN2*. Regrettably, in May 2024, Biogen and Ionis revealed that BIIB10 showed a notable decrease in *ATXN2* protein in the CSF but failed to demonstrate a reduction in NfL or any clinical advantage during the 6-month placebo-controlled period, leading to the halting of its development.

In a similar precision-medicine approach, a recent study explored using a human-derived monoclonal antibody (α-miSOD1) targeting misfolded SOD1 as a potential therapeutic strategy for ALS. This antibody selectively binds to misfolded SOD1 without interfering with its normal function. In preclinical models, administering α-miSOD1 successfully delayed disease onset, reduced motor neuron degeneration, and extended survival in *SOD1*-mutant ALS mouse models. Furthermore, the antibody detected misfolded SOD1 in postmortem spinal cord tissue from both fALS and sALS cases, highlighting its potential as a therapeutic agent for a broader patient population [48].

Another pharmacological strategy targeting *SOD1* involved the use of oral pyrimethamine, an FDA-approved medication used for the treatment of malaria and toxoplasmosis; in the clinical study, NCT01083667 demonstrated that pyrimethamine significantly lowered cerebrospinal fluid (CSF) *SOD1* levels in ALS patients with *SOD1* mutations, with a mean reduction of 13.5% at 18 weeks and 10.5% at 36 weeks. The treatment was well tolerated, and this trial marked a milestone as one of the first-ever ALS studies where patient genotype (*SOD1* mutation) and disease mechanism (gain-of-function of mutant *SOD1* protein in a dose-dependent manner) were used to guide therapy selection. These findings suggest pyrimethamine as a potential therapeutic approach for ALS patients with *SOD1* mutations [38].

A novel approach using ASO therapy aims to increase Stathmin5 expression in sALS patients, enhancing nuclear TDP-43 content and counteracting TDP-43 nuclear depletion, a hallmark of ALS pathology. A pilot AAV9-siRNA study also targeted *SOD1* in ALS patients with A4V and D90A mutations [56]. This groundbreaking study laid the foundation for a larger, ongoing gene therapy initiative to treat *SOD1*-ALS. The investigational gene therapy, AMT-162, uses an AAVrh10 vector to deliver a microRNA designed to knock down the expression of the mutated SOD1 protein. Preclinical studies in a *SOD1*-ALS mouse model showed that AMT-162 significantly improved survival and reduced SOD1 levels in spinal cord motor neurons. Further, reductions in SOD1 protein expression were observed in non-human primates at the proposed clinical doses. In October 2024, the first patient was dosed in the Phase I/II clinical trial of AMT-162, marking a key milestone in the development of this promising gene therapy for SOD1-ALS [80].

While much of the focus in ALS therapy development has been on *SOD1* mutations, another promising therapeutic approach is emerging for ALS patients with *FUS* mutations. The pilot study explored ION363 (also known as Jacifusen or Ulefnersen), an ASO targeting *FUS* mutations in ALS [36]. Results showed ION363 significantly reduced FUS protein levels in a *FUS-*ALS mouse model, preventing motor neuron loss and offering promise for disease progression reduction. The NCT04768972 trial is now evaluating ION363 in ALS patients with FUS mutations, with early data suggesting dramatic effects, particularly in younger patients, with some showing improvements in ALSFRS-R scores [36].Therefore, proper classification of the pathogenetic variants associated with ALS and a study to understand the harmful molecular mechanisms causing the disease are fundamental steps to develop new specific therapeutic strategies for this fatal disease.

### MiRNAs as biomarkers in ALS

The non-coding regions represent a large percentage of the human genome. [65] Among the different types of non-coding elements in the genome, there is growing evidence implicating microRNAs (miRNAs) in the pathogenesis of several neurodegenerative diseases, including FTD and ALS [35]. miRNAs directly interact with partially complementary target sites in target mRNAs’ 3′ UTR and repress their expression [33] and they play essential roles during differentiation and development. More than 60% of all mRNAs are estimated to contain miRNA target sites in their 3′ UTR, suggesting tight regulation and involvement in normal cellular homeostasis and disease states [70]. Furthermore, several studies show that various miRNAs can target a massive amount of mRNAs, suggesting the involvement of these small ncRNAs in the development of a multitude of diseases [40, 23], including several types of cancer [40], heart diseases such as hypertrophy and ischemia [70, 41] as well as neurodegenerative disorders (NDs) [69].

Diagnosing ALS primarily relies on a clinical assessment of a patient’s symptoms, which means observing the progressive spread of both upper and lower motor neuron signs across various body regions, as there is currently no single definitive test to confirm the disease, and diagnosis often occurs after significant motor neuron loss has already taken place [75]. Thus, for a drug to be effective, early or prodromal diagnosis would be necessary to prevent further motor neuron degeneration and to preserve the function of remaining motor neurons. However, this is challenging because no reliable molecular biomarkers have been identified in clinical trials for presymptomatic diagnosis or patient stratification. Several efforts are being made from this point of view. Recent studies have identified neurofilament light chain (NfL) and phosphorylated neurofilament heavy chain (pNfH) as reliable biomarkers for the disease. Notably, plasma NfL concentrations can increase 6 to 12 months before symptoms of ALS appear, making it a promising tool for early diagnosis [82]. Furthermore, measuring the enzymatic activity of *SOD1* has proven beneficial in ALS drug trials and large-scale screenings for *SOD1* mutations, providing a cost-effective alternative to next-generation sequencing [29]. The genetic landscape of ALS is slowly evolving in response to novel genetic discoveries, helping to identify pathogenic cellular pathways and providing potential biomarkers and targets for drug discovery [10].

miRNAs have been found to be excellent candidates as biomarkers for the disease. According to the National Institutes of Health Biomarkers Definitions Working Group, a biomarker is “*a characteristic that is objectively measured and evaluated as an indicator of normal biological processes, pathogenic processes, or pharmacologic responses to a therapeutic intervention*” [6]. In ALS cases, biomarkers would allow an earlier and more precise diagnosis, with the possibility to start treatments earlier to modify the disease course. These biomarkers could aid in the classification/stratification of ALS patients, in monitoring disease progression, and identifying patients who will respond better to a particular drug. Biomarkers can also provide a valuable tool for identifying new therapeutic approaches and driving patients’ enrollment in clinical trials. Furthermore, they may represent a link between the results obtained in animal models and human patients, providing insight into potential therapeutic targets.

Since miRNA constructs have a tissue-specific expression, it is possible to use differential expression in distinct tissues for various purposes. For example, circulating miRNAs can be used as predictive and/or diagnostic biomarkers. The use of blood samples in routine diagnostic testing presents several advantages. Blood specimens are easy to obtain, process, and store, and the samples required for the analysis can be collected without using invasive procedures for the patients. Furthermore, blood-based biomarkers may originate from the CNS through a transfer between the blood and CSF at the blood–brain barrier [34, 72], suggesting that the same biomarkers could be present in both biofluids. They may also be generated by other organs and tissues affected in ALS, such as degenerating muscles or peripheral blood cells. Therefore, blood can represent an excellent biofluid for discovering and validating biomarkers for ALS [83]. On the other hand, miRNAs present in blood can reflect other concurrent pathophysiological conditions that may not be directly related to ALS disease (e.g., inflammatory status, response to pharmacological treatments, etc.), which may complicate the interpretation, specificity, and utility of the biomarker in question.

Several studies highlighted how altered biogenesis and expression of miRNAs can be responsible for the degeneration of spinal motor neurons, both in fALS and sALS. In addition, miRNAs have been found to exert a function in neuronal inflammation in ALS. Numerous works showed that miRNAs in affected subjects could cross the blood–brain barrier to reach the bloodstream and, thereby, could be utilized as disease biomarkers. Highly deregulated miRNAs have been associated with different degrees of disease progression. miR-151a-5p, miR-199a-5p and miR-423-3p were seen to be down-regulated in affected subjects, whereas miR-338-3p, miR-206 and miR-133a appeared up-regulated. Moreover, up-regulation of miR-199a-5p, miR-206, and miR-133a correlated with a better prognosis and a slower disease course. miR-338-3p is involved in ALS pathogenesis, not only in tissues directly related to the disease but also in the peripheral tissues such as blood, helping to evaluate the potential of such miRNAs as a novel class of genetic blood marker for sALS [13]. miR-199a-5p has been involved in the initial phase of ALS and found to be significantly down-regulated in the last phase of the disease. In contrast, miR-206 has been supposed to mitigate the disease’s progression by enhancing the joints’ regeneration at the neuromuscular level [20]. miR-335-5p was found to be downregulated in ALS patient serum, increasing oxidative stress, inhibiting the caspase 3/7 apoptotic pathway, and deregulating neuronal degeneration [14, 69].

Recent studies have highlighted the presence of connections between miRNAs and RNA-binding proteins (RBPs), such as *TARDBP* and *FUS*, with essential regulatory complexes such as Drosha in the nucleus and Dicer in the cytoplasm. Drosha complexes with DGCR8 have also been associated with *TARDBP*, suggesting more complex dynamics than miRNAs and protein-related pathologies, especially in MNs. *FUS* localizes together with *TARDBP* in the Drosha nuclear complex, and the direct binding of *FUS* to the nascent pri-miRNAs allows to recruit Drosha to transcriptionally active sites for further processing of the pri-miRNAs themselves. Furthermore, *FUS* has been shown to promote gene silencing through direct binding to specific miRNA and mRNA targets. Mutations in this gene also impair the function of the AGO2 protein in the miRNA-induced silencing complex (miRISC) [61, 69].

**Fig. 5:**
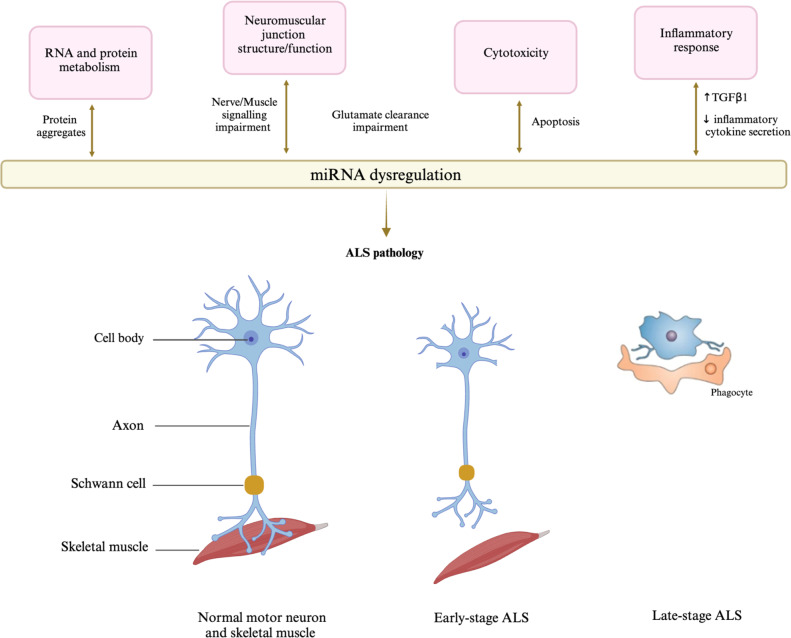
Dysregulation of miRNA biogenesis and function may contribute to the development and progression of ALS. Impairments in miRNA biogenesis or function occur through the dysregulation of different cellular pathways. Specifically, miRNA–protein complex metabolism can be modified by cytoplasmic protein inclusions, the transmission of wrongful cues owing to disrupted signaling at the neuromuscular junction, inhibiting of cell homeostasis due to cytotoxicity associated with faulty glutamate clearance, and an overactive inflammatory response. Conversely, research indicates that the malfunctioning of significant miRNAs initiates changes in these cellular pathways, leading to the downstream effects of the neurodegeneration linked to ALS pathology. (Created with BioRender.com)

The implications of miRNA dysregulation extend across diverse facets of ALS, including aberrant RNA and protein metabolism, inflammatory responses, cytotoxicity, and compromised neuromuscular junction structure and signaling (Fig. 5).

Considering all these evidences, it is necessary to promote the use of plasma miRNAs, in association with other biomarkers, to make predictive diagnoses and discover new therapeutic targets. There is still much work to be done to validate and demonstrate its clinical utility in the general ALS population. The ultimate goal is to include these biomarkers in all phases of ALS management, from diagnosis to clinical studies and, prospectively, to the identification of future therapeutic targets.

### LncRNA as potential therapeutic target

It is estimated that approximately 40% of lncRNAs are uniquely expressed in the brain, surpassing the number of protein-coding genes [18]. LncRNAs demonstrate tissue-specific expression and have a modulatory effect on the CNS by influencing various biological processes such as epigenetic modulation, post-transcriptional and translational regulation, alternative splicing, and cell cycle [89]. These molecules in brain tissue are comparatively more stable than those in other tissues. Furthermore, the lncRNAs in the brain display enhanced temporal and spatial specificity compared to mRNAs [63]. GWAS and comparative transcriptomic studies have associated lncRNAs with different NDs, including the age-onset motor neurons-associated disease ALS [49, 90]. However, most of these studies have described associations but do not show unequivocal evidence of causation.

**Fig. 6:**
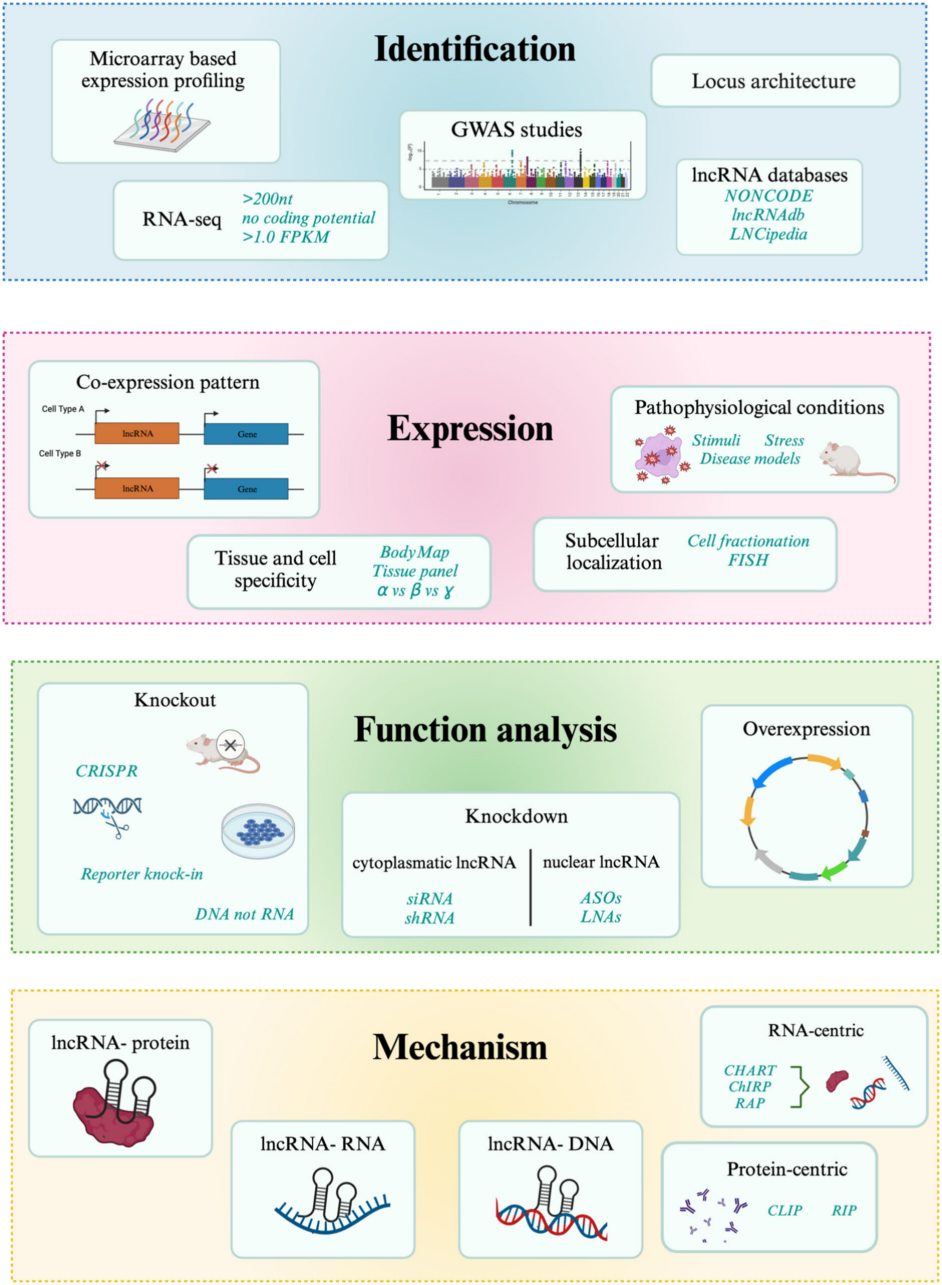
Overview of lncRNA discovery and characterization. (Created with BioRender.com)

Over the past few years, much experimental evidence has emphasized the role of lncRNAs in motor neuron development. During motor neuron development, the expression of both linear and circular lncRNAs increases. These RNA molecules are conserved across species and are involved in the recruitment and function of polycomb repressive complex 1/2. Interestingly, the same cluster of lncRNAs regulated during motor neuron development is often dysregulated in a motor neuron disorders context (e.g., *Lhx1os*, *lncMN-1*, *lncMN-2*). These results suggest that lncRNAs are crucial for normal motor neuron development and that their dysregulation could underlie the disease’s pathogenesis of ALS and SMA. The main challenge in the field is to identify ‘key’ downstream targets that are affected by lncRNA dysregulation and lead to motor neuron degeneration. Future work should focus on downstream targets beyond the direct interactors of lncRNAs, but to genes or proteins in extended molecular networks that may be involved in motor neuron degeneration. Developing innovative genetic tools and in vitro*/*in vivo models would be essential to address how dysregulated lncRNAs can cause disease. An approach is to take advantage of the iPSC and genome editing technology to generate patient-derived models (and isogenic controls) to dissect the different roles of lncRNAs. Knockdown approaches, co-immunoprecipitation, and argonaute 2-CLIP experiments combined with proteomics and next-generation sequencing analysis of lncRNAs are part of the toolbox that could reveal the nature and consequences of lncRNA interactions (Fig. 6).

Such experimental strategies would allow the establishment of a direct link between lncRNA-related targets and functions and consequent phenotypic alterations in motor neurons. An interesting hypothesis that needs further investigation is the possibility that lncRNAs may have a role to play in conferring selective vulnerability and degeneration of specific subtypes of motor neurons. Improving our understanding of the mechanism of action and roles of lncRNAs will stimulate generation of new ideas or molecular strategies for treating motor neuron diseases.

Given the ability of lncRNAs to regulate the NDs development through modulating different cellular mechanisms, such as autophagy, targeting autophagy-associated lncRNAs in neuronal cells may be a viable potential therapeutic strategy for treating these disorders. Multiple lines of evidence demonstrate that autophagy is critical for the homeostasis maintenance in neuronal cells [60, 46]. Furthermore, autophagy dysregulation is one of the main etiologies of NDs; thus, autophagy can be a promising therapeutic direction for treating NDs [17].

Currently, two major oligonucleotide-based strategies, ASOs and RNAi, have successfully been used to reduce the expression of upregulated lncRNAs in neuronal cells, demonstrating the vast therapeutic potential of RNA-based therapies for NDs [62, 66]. However, the main obstacle for delivery of these oligonucleotides into the central nervous system is the lack of transfer across the blood–brain barrier. Recent studies suggest that combining RNA-based therapies with liposomes can enhance blood–brain barrier penetration [26]. Cell-derived exosomes are also being considered as therapeutic vehicles to deliver RNAs to the CNS. A secondary hurdle to consider when targeting lncRNAs is the presence of secondary structure that is inherently encoded in its sequence which may decrease efficacy or block the binding of the ASOs or siRNAs to its target. However, this could be resolved by using chemically modified analogs of the oligonucleotides [5, 87]. Another important consideration is the availability of preclinical animal models of the disease for developing relevant therapies due to a lack of conservation between human and non-primate lncRNA sequences [51].

Currently, 11 RNA therapies are approved by the US Food and Drug Administration (FDA) and/or the European Medicines Agency (EMA); of these, only one, Nusinersen, aimed at improving SMA outcomes, is targeted to the CNS via intrathecal administration [85]. Although no approved RNA therapies are available to target lncRNA molecules in humans, several experiments are being conducted in preclinical models.

Considering these new lines of investigations, the characterization of lncRNAs and their future use as therapeutic targets in ALS could strongly contribute to the therapy of such a devastating disease.

## Conclusion

In conclusion, through GWAS, NGS, and the identification of key genetic variants, significant progress has been made in unraveling the complex etiology of complex diseases, such as neurodegenerative disorders. Additionally, the emerging knowledge of miRNAs and lncRNAs as potential biomarkers and therapeutic targets offers interesting opportunities for early diagnosis and precision medicine for a devastating and fatal disease such as ALS.

However, it is essential to acknowledge the limitations and challenges that persist in this field. Issues such as blood–brain barrier penetration, the need for more accurate preclinical models, and the development of effective delivery systems for RNA-based therapeutics remain significant hurdles. Moreover, while genetics has provided valuable insights, neurodegenerative diseases are multifactorial, and environmental factors likely play a role that requires further exploration. To address these challenges and continue advancing research in this field, interdisciplinary collaboration and innovation are crucial.

## Data Availability

Not applicable.
